# Building Effective Machine Learning Models for Ankle Joint Power Estimation During Walking Using FMG Sensors

**DOI:** 10.3389/fnbot.2022.836779

**Published:** 2022-04-01

**Authors:** Oliver Heeb, Arnab Barua, Carlo Menon, Xianta Jiang

**Affiliations:** ^1^Biomedical and Mobile Health Technology Laboratory, ETH Zurich, Zurich, Switzerland; ^2^Department of Computer Science, Memorial University of Newfoundland, St. John's, NL, Canada; ^3^Menrva Research Group, Schools of Mechatronic Systems Engineering and Engineering Science, Simon Fraser University, Burnaby, BC, Canada

**Keywords:** gait analysis, FMG, ankle joint power, machine learning, LSTM, CNN

## Abstract

Ankle joint power is usually determined by a complex process that involves heavy equipment and complex biomechanical models. Instead of using heavy equipment, we proposed effective machine learning (ML) and deep learning (DL) models to estimate the ankle joint power using force myography (FMG) sensors. In this study, FMG signals were collected from nine young, healthy participants. The task was to walk on a special treadmill for five different velocities with a respective duration of 1 min. FMG signals were collected from an FMG strap that consists of 8 force resisting sensor (FSR) sensors. The strap was positioned around the lower leg. The ground truth value for ankle joint power was determined with the help of a complex biomechanical model. At first, the predictors' value was preprocessed using a rolling mean filter. Following, three sets of features were formed where the first set includes raw FMG signals, and the other two sets contained time-domain and frequency-domain features extracted using the first set. Cat Boost Regressor (CBR), Long-Short Term Memory (LSTM), and Convolutional Neural Network (CNN) were trained and tested using these three features sets. The results presented in this study showed a correlation coefficient of *R* = 0.91 ± 0.07 for intrasubject testing and were found acceptable when compared to other similar studies. The CNN on raw features and the LSTM on time-domain features outperformed the other variations. Aside from that, a performance gap between the slowest and fastest walking distance was observed. The results from this study showed that it was possible to achieve an acceptable correlation coefficient in the prediction of ankle joint power using FMG sensors with an appropriate combination of feature set and ML model.

## Introduction

In gait analysis, ankle joint power can play an essential role to address the issues regarding abnormal gait actions (Zelik and Honert, 2018). For example, a decreased ankle joint power indicates muscle weakness and a diminution of health. However, measuring the ankle joint power is still complex and expensive, which may involve setting up a special treadmill, a motion tracking system, and an individual biomechanical model (Zelik and Honert, 2018). Therefore, in recent studies, attempts have been made to facilitate this process by eliminating the need for a complex biomechanical setup.

An approach to facilitate ankle joint analysis was made in Zheng et al. (2008). In this study, a force sensor in the shoe measures the ground reaction force while three Inertial Measurement Units (IMUs) detect the lower limb movement. The ankle joint power was obtained using the inverse kinetics method. In further research (Miyashita et al., 2019), a single IMU is used to estimate the ankle joint power by regression analysis. However, the studies solely estimated the ankle joint power during the terminal stance phase. Another example to assess the ankle joint power for the complete gait phase is presented in Jiang et al. (2019) and Barua et al. (2021). Data from IMUs combined with divergent signal processing techniques and machine learning (ML) models have already been employed to achieve a considerable performance in the prediction of ankle joint power.

However, the performance of other sensors, such as force myography (FMG) using force resisting sensor (FSR) sensors for similar applications, is yet to be explored. FSR sensors measure the volumetric changes of the underlying muscles through pressure (Xiao and Menon, 2019). The advantages of FMG include eliminating the alteration of data due to sweating when compared to surface electromyography (Victorino et al., 2018). Moreover, FSR sensors are lightweight and cost-effective (Jiang et al., 2017a). Therefore, FMG sensors carry potential significance for the welfare of healthcare using wearable technologies (Xiao and Menon, 2019). The effectiveness of FMG sensors has already been explored in various fields of health technology, such as hand gesture recognition (Jiang et al., 2017b; Asfour et al., 2021), prostheses or orthoses control (Xiao et al., 2014; Ahmadizadeh et al., 2019), hand force estimation (Sakr and Menon, 2018), and gait phase classification (Chu et al., 2017; Jiang et al., 2018a,b). In these studies, FMG sensors provided time-series muscle contraction signals, which were then processed with ML algorithms for divergent applications.

Machine learning techniques have been immensely popular in the last few decades because of their incredible ability to recognize patterns and create complex relationships in data. Besides, advancement in deep learning (DL) techniques has introduced Long-Short Term Memory (LSTM) and Convolutional Neural Network (CNN), which are well-known for their admirable capabilities to address the relationship among time-series data (Song et al., 2020) and repetitive patterns by extraction of significant features (Kiranyaz et al., 2021).

Considering the discussion mentioned above, we present the feasibility of estimating the ankle joint power with ML techniques using FMG signals in this paper. To our knowledge, this is the first work that includes the usage of FMG signals in the prediction of ankle joint power by exploring appropriate combination among ML models and feature sets. We hypothesized that the ankle joint power could also be estimated by FMG signals and receive a similar accuracy as the IMU-based estimation (correlation coefficient > 0.90) (Jiang et al., 2019) with a correct fusion of features and ML model. The rest of the paper is structured as follows: Section Materials and Methods describes the data accumulation, the signal processing steps, and the ML algorithms. Section Results presents the result and comparative evaluation. Finally, the results are discussed in Section Discussion and we concluded the outcomes in Section Conclusion.

## Materials and Methods

**Figure 1:**
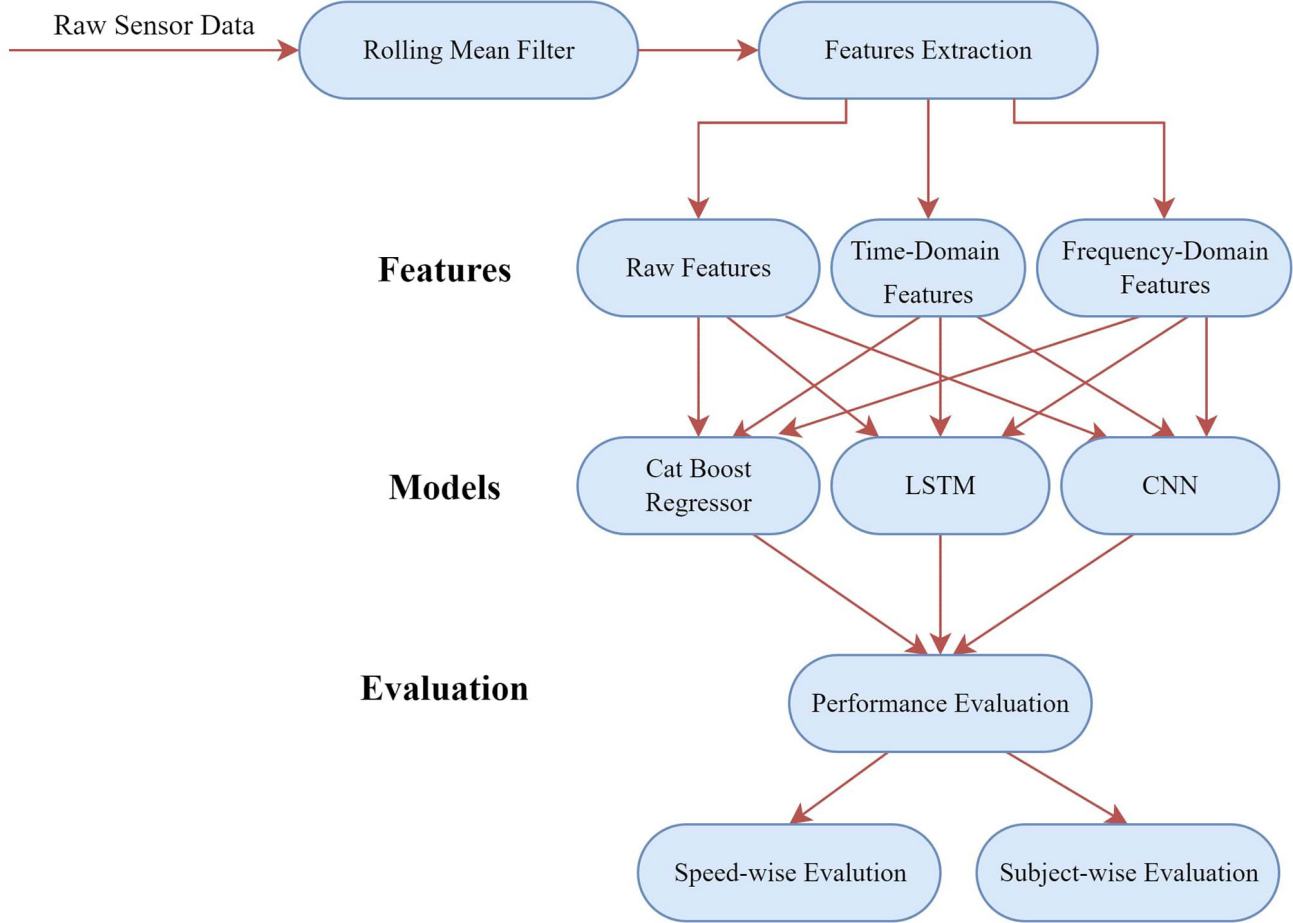
An overview of the signal processing steps conducted in this study.

A pictorial view of the overall procedure is presented in Figure 1, which depicts the workflow of this whole study. It should be mentioned that the data analyses, preprocessing steps, and the ML models were implemented using Python 3.7.10 in Google Colaboratory environment with the packages sklearn, keras, and scipy.

### Data Accumulation

The data used in this study were collected from nine young, healthy male participants. [avg. height: 76 ± 7 cm, avg. weight: 72 ± 9 kg, avg. age: 27 ± 8] in a controlled environment (Jiang et al., 2019). The Office of Research Ethics at Simon Fraser University approved the study protocol, and all participants provided informed consent. More details of the experiment and the study protocol can be found in Jiang et al. (2019).

Before the experiment, the participants were equipped with an FMG strap that consisted of 8 FSR sensors (FSR 402) (Interlink Electronics Inc., Los Angeles, CA, USA) (Jiang et al., 2018a). The FMG strap was positioned around the participant's right leg about 2 inches above the ankle (Jiang et al., 2018a). Besides, a Vicon motion capture system was installed for capturing the movement of the 14 reflective markers that were placed on the thigh, shank, and foot of the left lower limb. A force-plate-instrumented treadmill was used to measure the vertical ground reaction force as a final tool for the experiment. For the detailed experiment setting, please see Jiang et al. (2019). During the experiment, each participant walked for a minute at each of the 5 divergent velocities (0.4, 0.7, 1.0, 1.3, and 1.6 m/s) on the force-plate-instrumented treadmill. Meanwhile, the data from the FMG strap, the force-plate-instrumented, and the motion capture system were sampled synchronously with a sampling frequency of 100 Hz and accumulated for offline processing. The ground truth value (the ankle joint power) was calculated by a biomechanical model, with further processing using the vertical ground force and the value from the Vicon motion capturing system (Jiang et al., 2019). This process to determine the ankle joint power is the gold standard (Bogey et al., 2010). The arrangement mentioned above led to around 30,000 data points for each participant.

While analyzing the participants' data, abnormalities in the reference value (ankle joint power) were detected. The reference value consisted of non-logical high or low values for some data points. In this case, we set maximum and minimum boundaries, which were decided by observing the values of the regular maximum and minimum peaks.

### Preprocessing

**Figure 2:**
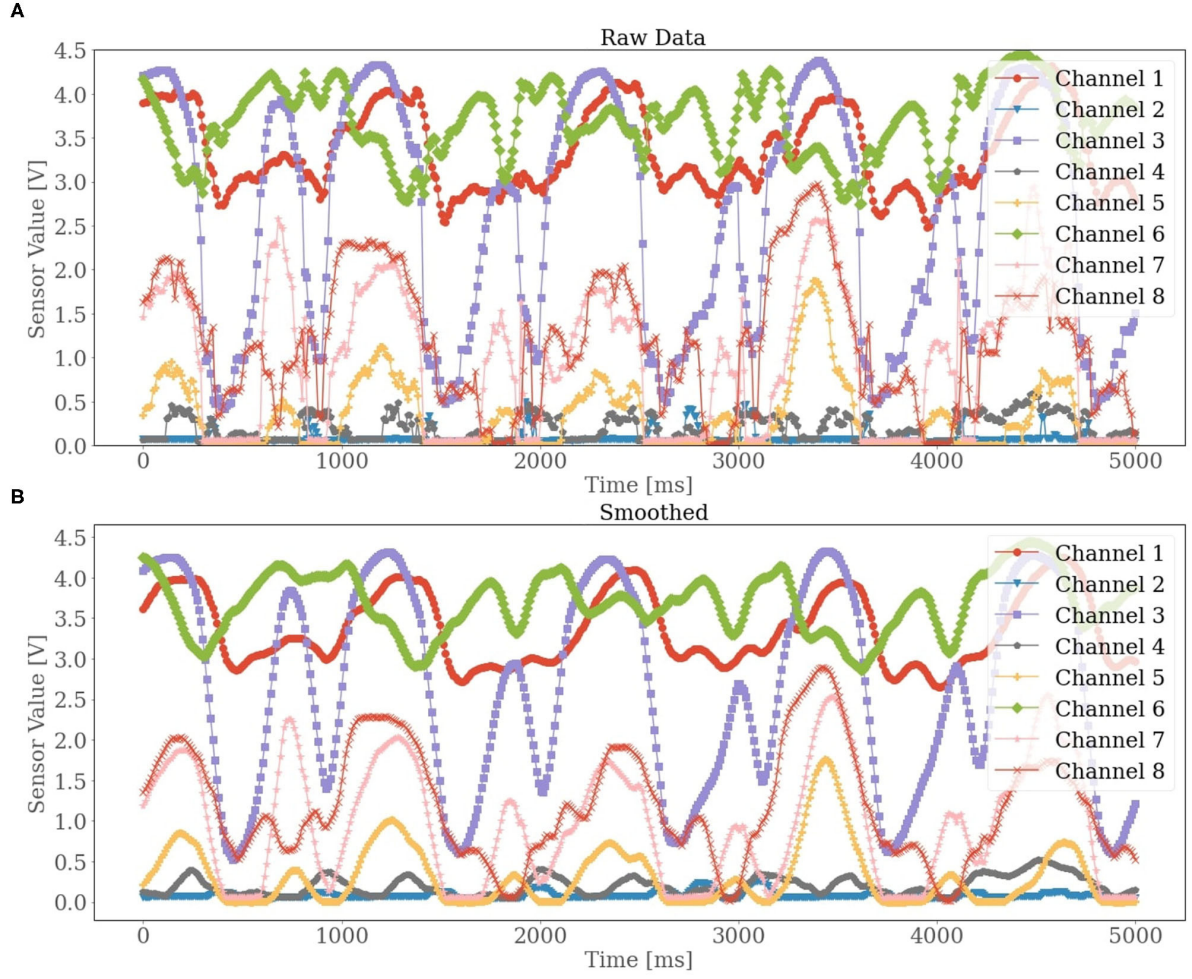
A given example of the raw data collected from the force myography (FMG) strap. The 8 channels correspond to the 8 force resisting sensor (FSR) sensors, respectively. In diagram **(A)**, the raw unfiltered sensor values are presented, while in diagram **(B)** the smoothed sensor values are displayed.

The 8 FSR sensors on the FMG strap provided 8 channels of a time-series signal. Each channel describes the muscle contraction in a different strap position around the lower leg. To smooth out short-term fluctuation in the raw signal, a rolling mean filter with a window size of 120 ms and an overlapping of 110 ms was applied. The averaged value from each window corresponds to the reference value of the last row of the corresponding window. In Figure 2, two plots of the raw and filtered signals are shown to depict the elimination of short-term fluctuation after smoothing. After filtering, we performed a min-max normalization. In ML algorithms, the normalization of the data may help to improve the performance. Especially for gradient descent-based algorithms or Neural Network (NN), downscaling the data to a smaller range assists the network in converging more quickly toward the minima. Keeping this in mind, we employed a min-max scaler to scale the features in a range between 0 and 1. Additionally, a min-max scaler also normalized the target variable on a range between −1 and +1. The predicted ankle joint power made by the model was later transformed inversely before computing the evaluation metrics.

### Feature Extraction

To facilitate the prediction of ankle joint power, we decided to use time-domain and frequency-domain features for their well-known significance in the improvement of the performance of ML models.

The time-domain features are used in signal regression due to their easy and quick implementation. Besides, the time-domain features were seemed to be considered applicable when dealing with FMG signals (Barioul et al., 2020). From Altin and Er (2016), we decided on using 7 features, which are presented in Table 1. We sampled the data into the sliding window of length 120 ms and allowed 110 ms overlapping between two consecutive windows to extract the features. At this moment, the window size of 120 ms was found empirically. In total, 56 features were extracted for the 8 channels.

**Table 1:** Formulas of the time-domain features.

**Time domain features**	**Formula**
Mean	$mean=\;\frac{1}{N}\overset{N}{\underset{n=1}{\sum }}x_{n}$
Standard deviation	$std=\;\sqrt{\frac{1}{N-1}\overset{N}{\underset{n=1}{\sum }}{\left(x_{n}-\;\mu \right)}^{2}}$
Variance	$var=\;\frac{1}{N-1}\overset{N}{\underset{n=1}{\sum }}{\left(x_{n}-\;\mu \right)}^{2}$
Maximum	$\max=\;\underset{n\in N}{\max}\;x_{n}$
Minimum	$\min=\;\underset{n\in N}{\min}\;x_{n}$
Skewness	$skew=\;\frac{\frac{1}{N}\overset{N}{\underset{n=1}{\sum }}{\left(X_{n}\;-\;\mu \right)}^{3}}{\sigma^{3}}$
Kurtosis	$kurt=\;\frac{\frac{1}{N}\overset{N}{\underset{n=1}{\sum }}{\left(X_{n}\;-\;\mu \right)}^{4}}{\sigma^{4}}$

For extracting frequency domain features, we used the Fast Fourier transformation (FFT), an algorithm that computes the discrete FT and converts the signal from the time-domain into discrete frequencies in the frequency domain (Faraggi and Sayadi, 2019). For the FFT, the Hanning window was preferred due to its less spectral leakage (Lyon, 2009). The data were segmented into overlapping (95%) windows where each window contained data corresponding to 200 ms. Here, the window size was also found by trial and error. The extracted data point from each window corresponded to the ankle joint power of the last row of that corresponding window. Then the FFT transformation was applied to each channel from the signal decomposing the time signal into discrete frequencies. After transforming, solely magnitude of the two primary frequencies was extracted and later used as features because for higher frequencies, the magnitude was near zero. Extracting the two primary frequencies from the 8 channels, the FFT resulted in 16 features for each time step.

### ML Models

We decided to use LSTM as our first predictive model, considering the characteristics of the time-series data in the accumulated dataset. The LSTM, a type of Recurrent NN, is powerful to handle time-series data (Song et al., 2020). It has feedback connections when compared to other NNs, which allows it to save past information within the internal memory. The ability to learn from past data points makes the LSTM powerful in handling cycled time series. The LSTM model we designed was based on the paper (Barua et al., 2021). The model consisted of an LSTM layer with 1,024 neurons and a tanh activation function followed by three dense layers with 256, 128, and 64 neurons, respectively. Each layer had a relu activation function. The last layer was a dense layer that consisted of only one neuron and a linear activation function to predict the target variable.

We employed 1-D CNN as our second predictive model. Compared to LSTM, CNN is popular for image or language processing (Kiranyaz et al., 2021). However, the 1-D CNN allows using the model for time-series data by identifying the repetitive patterns in the data. Besides, the 1-D CNN model can perform convolution operations to extract high-level features through divergent filters. Therefore, a CNN model requires less preprocessing and can directly apply to raw signals. The CNN model design was also based on Barua et al. (2021). We initiated the CNN model by introducing three successive convolution layers with kernel sizes of 7, 5, and 3 and filter sizes of 1,024, 512, and 256, respectively. We added two dropout layers with a dropout probability of 0.3 among the first three convolution layers (Barua et al., 2021). The goal of these dropout layers was to avoid overfitting. The convolution layers were followed by a 1-D average pooling layer and a dense layer that contains 128 neurons. A dense layer with one neuron and a linear activation function were defined for the output layer. Finally, for all the other layers except for the first one, a relu activation function was used, while for the first layer we used a tanh activation function. The reason behind choosing LSTM and CNN as predictive models to predict the ankle joint power was their superior performance, which is presented in Barua et al. (2021), using a dataset of similar volume we used in this study.

Additionally, we used the Cat Boost Regressor (CBR) model to observe the performance of a conventional ML model in a similar task. The reason behind choosing a boosting model was its extraordinary capability to create a strong learner by sequentially aligning weak learners. CBR, belonging to the class of boosting models, uses gradient boosting on decision trees (Dorogush et al., 2018). We only tuned two significant parameters of CBR, namely, max depth and max iterations. We set the max depth to 3 to avoid overfitting and increased the iterations to 2,000 for a more precise prediction. Equally to the NN, we defined mean squared error (MSE) as the loss function.

### Performance Metrics

Metrics are measures commonly used in ML to evaluate predictive performance. For our study, we decided on using the following 3 popular performance metrics in regression:

#### Correlation Coefficient (*R*-value)

The correlation coefficient, also known as the *R*-value, produces a value between −1 and +1 to quantify the strength of dependencies between two variables. As the *R*-value goes closer to +1, we are expected to achieve a high closeness measurement between the predicted and true values.

#### Root Mean Squared Error

RMSE refers to the standard deviation (SD) of the error residuals between reference value and predictions. By squaring the error, the RMSE gives more weight to higher errors. RMSE is a negatively oriented metric, which means that a small RMSE value is desired. It is formulated using the following equation.


$$RMSE=\sqrt{\frac{1}{N}\sum {\left(reference\;value-predicted\;value\right)}^{2}}\;$$

#### Mean Absolute Error

MAE is also a negatively oriented metric and measures the average amount of residual error for the predictions. Compared to RMSE, the errors are weighted equally.


$$MAE=\frac{1}{N}\sum |reference\;value-predicted\;value|\;$$

### Validation Procedure

#### Data Reshaping

To feed the dataset into the mentioned DL models, we had to reshape the data into overlapping windows, each containing a fixed number of consecutive data points. For this study, the empirically chosen window size of 150 ms was used with an overlapping of 142 ms. Ankle joint power belonging to the last row of each window acted as the dependent data point for that corresponding window. Here, the windowing is performed after splitting the data into train and test sets, such that there is not a risk of data leakage. It should be mentioned that we trained the models for 40 epochs with an early-stopping technique and used Adam as an optimizer with a fixed learning rate of 0.001 and MSE as a loss function.

#### Validation Techniques

**Figure 3:**
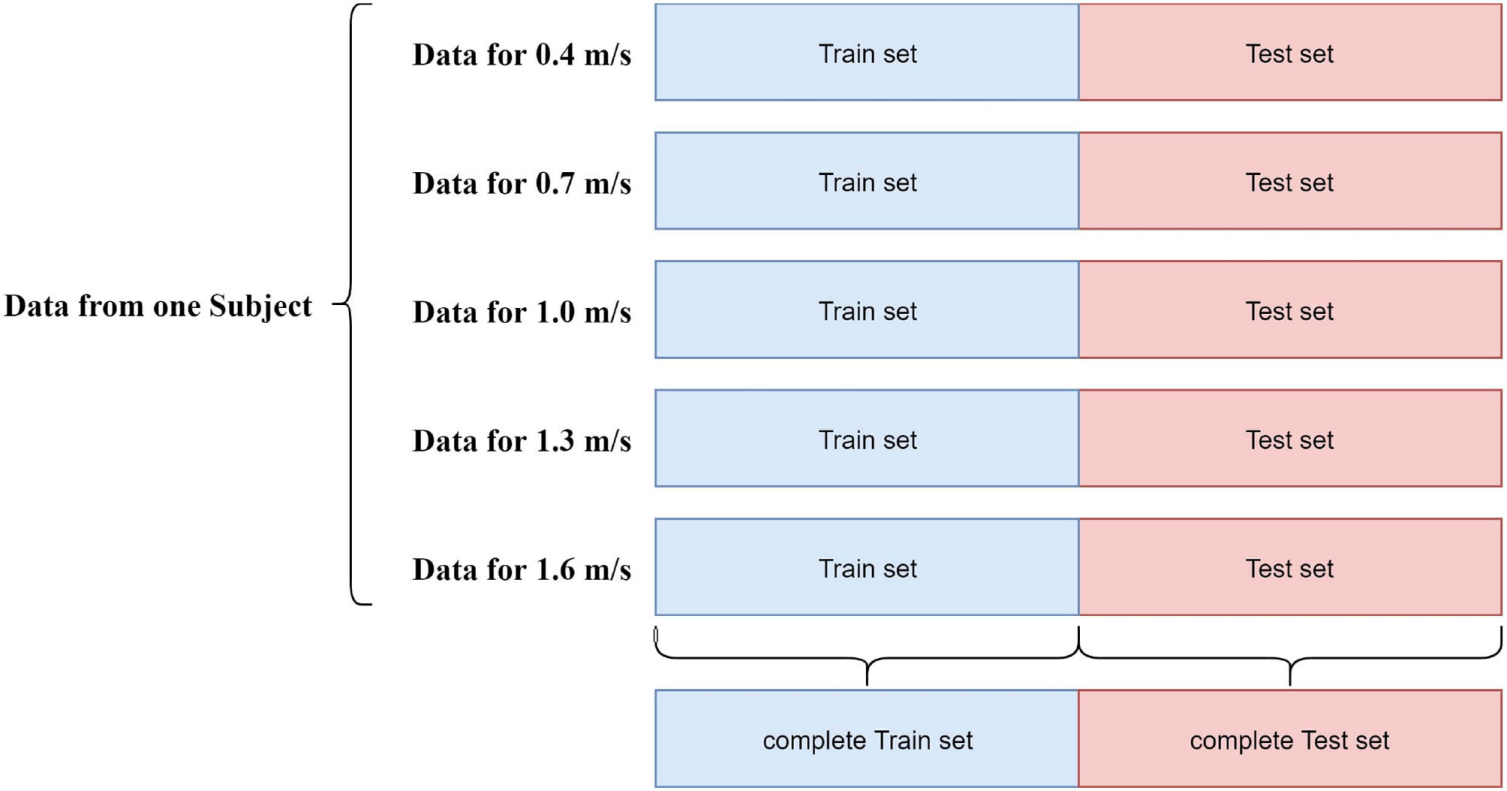
Overview of the data splitting procedure.

We performed three types of evaluation in our study. At first, we performed an overall evaluation. To conduct this, posterior to reshaping the data, we split the dataset for each participant into train and test set in the following manner. For each participant, we accumulated the first half (30 s) of data from each velocity to the train set, and the next half (30 s) was stored in the test set. A detailed overview of the data splitting process for overall evaluation is shown in Figure 3. The reason behind performing this 50/50 spilt was to ensure data from all types of velocities in both train and test sets. Following this manner, we acquired 9 train sets and 9 test sets corresponding to the 9 individual participants for a particular type of feature set. Since we considered 3 types of feature sets (raw channels, time-domain features, and frequency domain features) for this study, using the splitting manner mentioned above, we had 3 train sets and test sets for each subject corresponding to 3 different feature types. Therefore, we had 27 train sets and test sets, considering all 3 types of feature sets. We trained our aforementioned 3 models with a train set of each subject and evaluated the performance metrics using the test set of the corresponding subject. After completing the same validation procedure for all the subjects, we averaged the outcome of the evaluation metrics and computed the final performance metrics for each type of regressor. We achieved 3 different averaged outcomes corresponding to 3 different feature sets.

#### Speed-Wise Evaluation

After performing the overall evaluation, we conducted the speed-wise evaluation. We computed the performance metrics for each type of speed. We trained each model with the train set we made for overall evaluation but did not compute the performance metrics for the whole test set. Instead, we computed the performance metrics using the data of each velocity present in the test set separately for each subject. It was done to observe the performance of the models for each walking speed.

#### Subject-Wise Evaluation

Finally, the model's performance was also analyzed subject-wise, where the performance metrics were computed for the complete set of data for each subject. Such that, we were able to present the total evaluation metrics after averaging over the 9 subjects and analyzing the performance of the models for each subject individually. Here, the complete data for each subject were separated into 3-folds, with each fold containing a third of the data regarding each of the 5 velocities. The models are trained for each subject on 2-folds and tested on the third. Finally, we averaged the three metrics over the 3-folds. The reason behind using different evaluation procedures was to ensure that our proposed approach could produce equally effective performance in terms of a divergent evaluation scheme.

## Results

### Speed-Wise Evaluation

**Figure 4:**
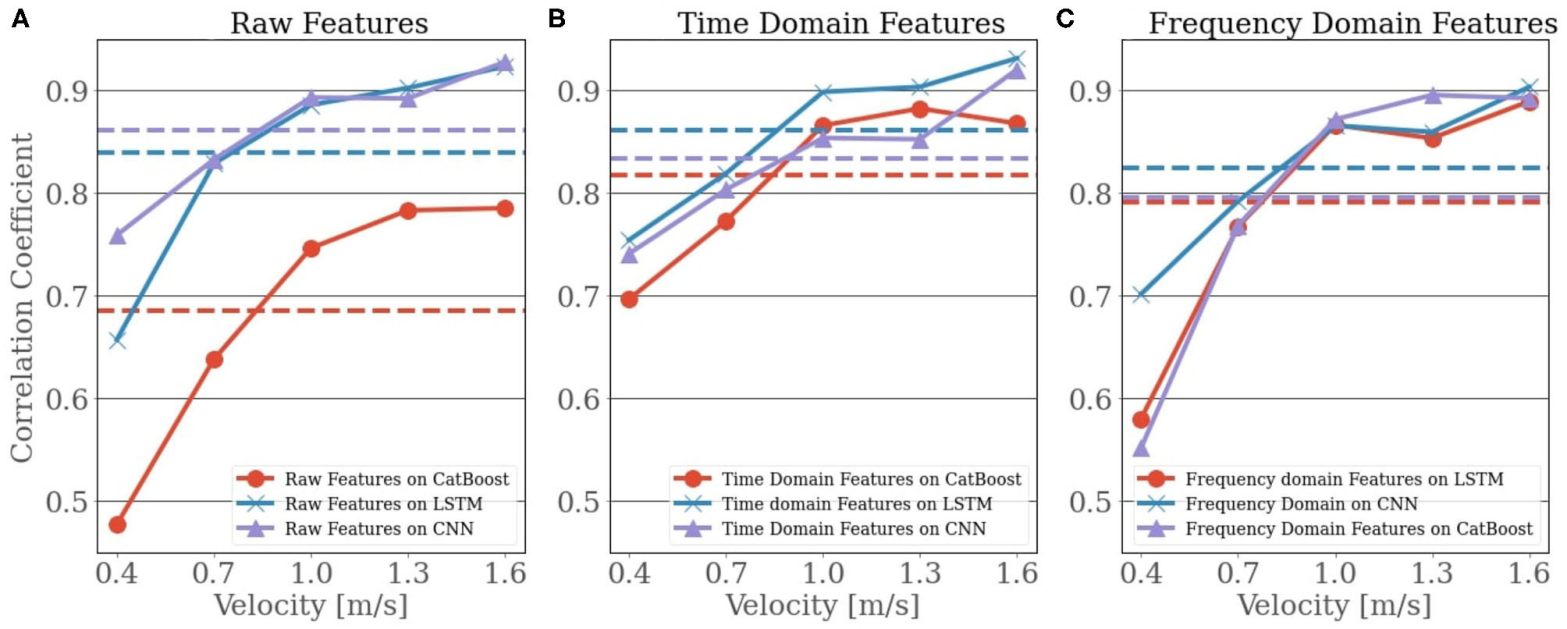
The plot shows the speed-wise correlation coefficient for the three models, Cat Boost Regressor (CBR), Long-Short Term Memory (LSTM), and Convolutional Neural Network (CNN), with each being separated into the three feature sets. The dashed lines represent the average over the velocities. In **(A)** the raw signal was used as predictors, where in **(B)** used the extracted time-domain features and in **(C)** the extracted features in frequency domain were used.

Although we evaluated each model for every type of feature set, we presented the outcome for the models that performed the best for each feature set elaborately to reduce the complexity of comparison. We found that CNN outperformed other models when we used raw feature set and frequency domain feature set. For time-domain features, LSTM had the upper hand. However, to support the choice of the best models for the different feature sets, we provided a pictorial view in Figure 4 that depicts the comparison between the outcomes of all 3 models for all 3 feature sets. From Figure 4, it can be observed that the CNN (*R* = 0.86) outperformed both CBR (*R* = 0.68) and LSTM (*R* = 0.84) when raw features were used. However, the difference between the outcome of CNN and LSTM was not highly significant. Although CBR could not deliver a considerable outcome using raw features, it showed a steady performance similar to LSTM and CNN for both time-domain and frequency-domain features. For time-domain features, LSTM achieved an average *R*-value of 0.86, which was high enough to outperform CNN (*R* = 0.83) and CBR (*R* = 0.81). For the frequency domain features, CNN (*R* = 0.82) showed slightly better performance when compared with the outcomes of LSTM (*R* = 0.79) and CBR (*R* = 0.79). In short, CNN was observed to be an appropriate choice when using raw and frequency domain features, whereas LSTM seemed to be more impactful in the case of time-domain features. However, considering the whole picture, CNN with raw features and LSTM with time-domain features should be preferable to frequency-domain features. An in-depth analysis of the best combination of the feature sets and models is presented in Table 2.

**Table 2:** The averaged metric values over the 9 participants for the Long-Short Term Memory (LSTM) and Convolutional Neural Network (CNN) on the three feature sets.

**Correlation coefficient**	**0.4 m*/*s**	**0.7 m*/*s**	**1.0 m*/*s**	**1.3 m*/*s**	**1.6 m*/*s**
CNN on raw features	0.76 ± 0.08	0.83 ± 0.11	0.89 ± 0.10	0.90 ± 0.10	0.93 ± 0.05
LSTM on time-domain features	0.74 ± 0.10	0.83 ± 0.11	0.89 ± 0.08	0.92 ± 0.07	0.92 ± 0.05
CNN on frequency-domain features	0.71 ± 0.11	0.76 ± 0.08	0.87 ± 0.05	0.85 ± 0.12	0.88 ± 0.07
**RMSE**	**0.4 m** ***/*** **s**	**0.7 m** ***/*** **s**	**1.0 m** ***/*** **s**	**1.3 m** ***/*** **s**	**1.6 m** ***/*** **s**
CNN on raw features	0.13 ± 0.04	0.22 ± 0.10	0.28 ± 0.15	0.39 ± 0.22	0.43 ± 0.17
LSTM on time-domain features	0.35 ± 0.05	0.46 ± 0.01	0.51 ± 0.13	0.60 ± 0.17	0.64 ± 0.13
CNN on frequency-domain features	0.60 ± 0.04	0.67 ± 0.07	0.71 ± 0.08	0.77 ± 0.10	0.8 ± 0.08
**MAE**	**0.4 m** ***/*** **s**	**0.7 m** ***/*** **s**	**1.0 m** ***/*** **s**	**1.3 m** ***/*** **s**	**1.6 m** ***/*** **s**
CNN on raw features	0.09 ± 0.02	0.13 ± 0.05	0.16 ± 0.07	0.21 ± 0.113	0.22 ± 0.08
LSTM on time-domain features	0.10 ± 0.04	0.15 ± 0.06	0.16 ± 0.07	0.20 ± 0.08	0.23 ± 0.10
CNN on frequency-domain features	0.09 ± 0.02	0.14 ± 0.05	0.17 ± 0.06	0.23 ± 0.01	0.26 ± 0.10

*The metrics are separated into different velocities*.

In Table 2, we presented the outcome of best models for corresponding feature sets for each type of walking speed. From Table 2, we can see a performance gap between the slower and higher velocities, especially in the frequency domain. We can observe that the performance of the models was decreased when predicting ankle joint power for slower speeds (0.4 and 0.7 m/s). For instance, if we consider LSTM on time-domain features, the correlation coefficient for the slowest velocity was 0.75, compared to the correlation coefficient for the highest velocity, 0.93. An identical picture can be observed for CNN with the frequency-domain features where it achieved an *R*-value of 0.70 for the velocity of 0.4 m/s and 0.90 for the velocity of 1.6 m/s. Additionally, we can see that the CNN on raw features has the highest correlation coefficient *R* = 0.76 for the slowest speed (0.4 m/s), and a correlation coefficient of *R* = 0.93 for the velocity of 1.6 m/s makes it a better choice for this kind of study.

The findings of the correlation coefficient are supported by the evaluation of the other two metrics, RMSE and MAE. It should also be noted that RMSE and MAE were increased with higher velocities while the accuracy was improved as the ankle joint power for higher velocities had a higher magnitude that resulted in a larger error residual.

**Figure 5:**
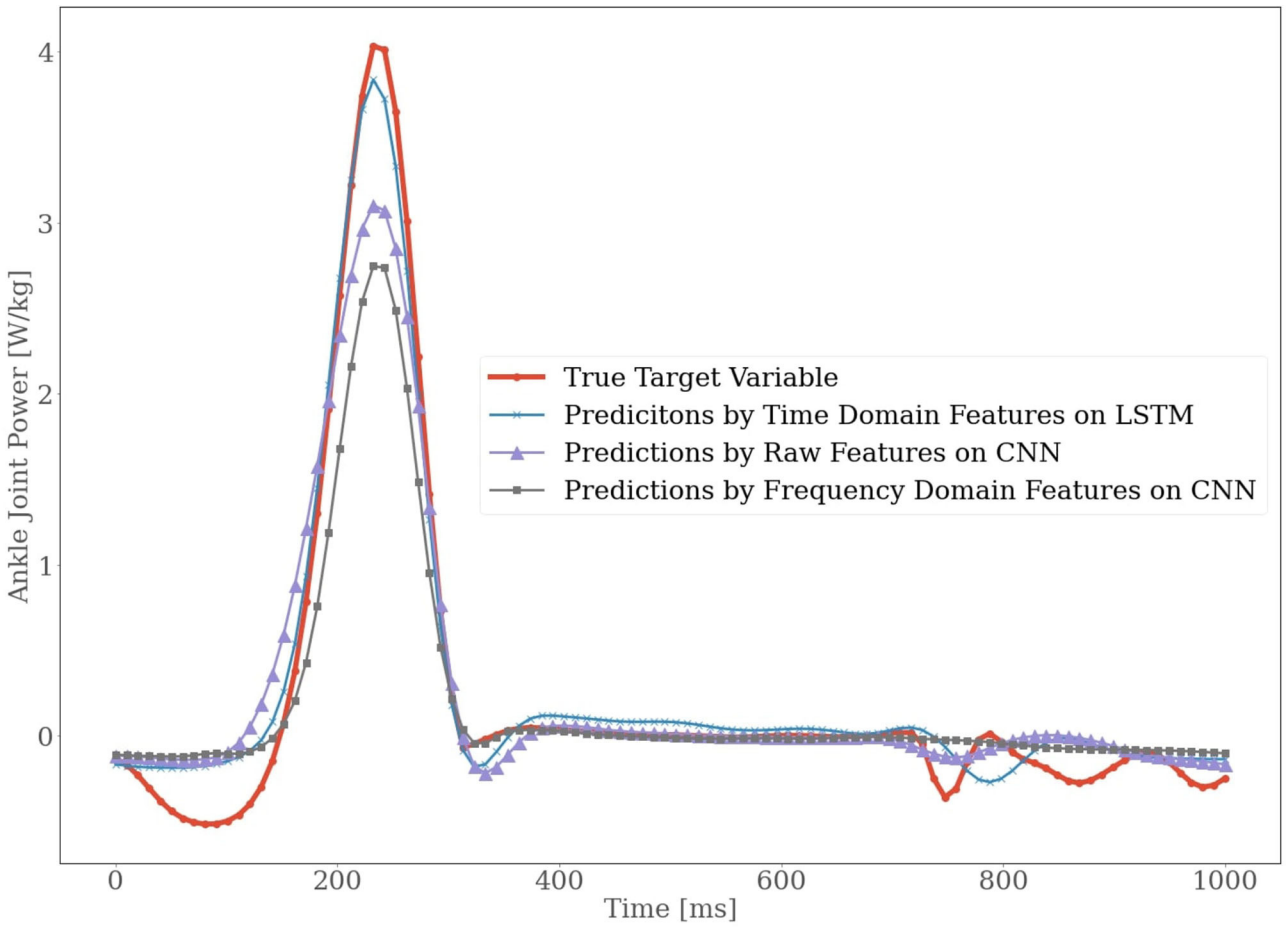
The True target variable is overlayed with the predictions from the two models Long-Short Term Memory (LSTM) and Convolutional Neural Network (CNN). This excerption presents participant 3 for the velocity of 1.6 m/s.

Another interesting thing we observed during our study is that although CNN on raw features performed better than other models, it still had difficulty in accurately predicting the peak ankle joint power. The same scenario was observed in the case of CNN on frequency domain features too. In Figure 5, a graphical view of the true ankle joint power vs. predicted ankle joint power is presented for subject 3 for the velocity of 1.6 m/s. We can see the suffering of all models in predicting peak values. A possible explanation is that the peak power value varies from cycle to cycle, making it harder to estimate the peaks correctly.

### Subject-Wise Evaluation

**Figure 6:**
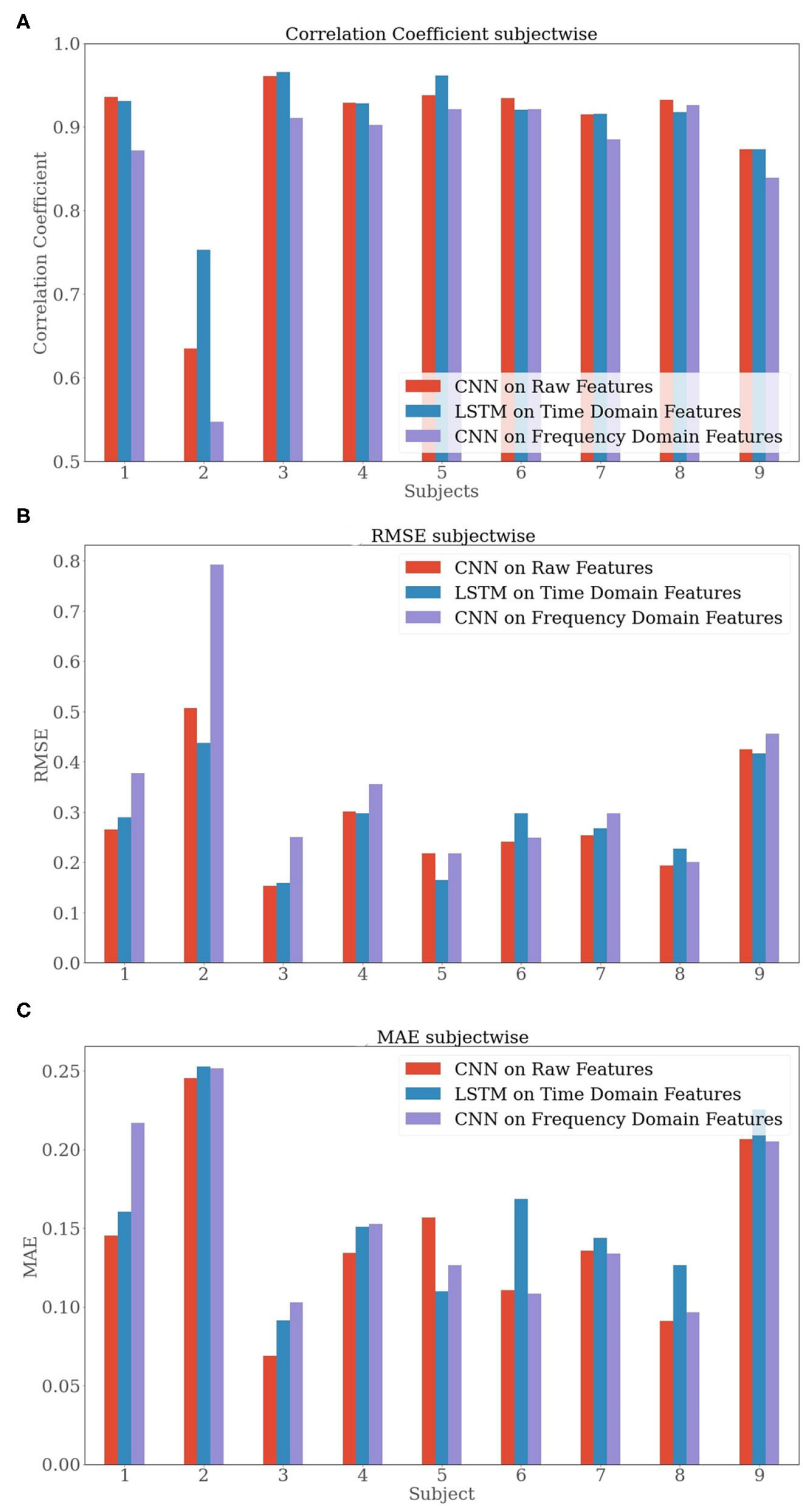
Bar plots of the subject-wise evaluation metrics **(A)** Correlation Coefficient, **(B)** RMSE, and **(C)** MAE) averaged over a 3-fold cross-validation.

As mentioned earlier, we used a 3-fold cross-validation technique where each fold contained a third of data from each type of 5 divergent velocities for each subject. The respective results are presented in Figure 6, Table 3.

**Table 3:** The subject-wise and averaged metric values of the 9 subjects for the Long-Short Term Memory (LSTM) and Convolutional Neural Network (CNN) on the three feature sets.

		**Raw features on CNN**	**Time-domain features on LSTM**	**Frequency-domain features on CNN**
Subject 1	R	0.94 ± 0.02	0.93 ± 0.02	0.88 ± 0.01
	RMSE	0.27 ± 0.02	0.29 ± 0.04	0.37 ± 0.05
	MAE	0.15 ± 0.004	0.16 ± 0.02	0.22 ± 0.05
Subject 2	R	0.64 ± 0.07	0.75 ± 0.02	0.55 ± 0.29
	RMSE	0.51 ± 0.05	0.44 ± 0.01	0.82 ± 0.05
	MAE	0.25 ± 0.02	0.25 ± 0.02	0.25 ± 0.05
Subject 3	R	0.96 ± 0.01	0.97 ± 0.01	0.91 ± 0.02
	RMSE	0.15 ± 0.01	0.16 ± 0.02	0.24 ± 0.06
	MAE	0.07 ± 0.001	0.09 ± 0.2	0.2 ± 0.09
Subject 4	R	0.93 ± 0.03	0.93 ± 0.01	0.90 ± 0.02
	RMSE	0.30 ± 0.06	0.30 ± 0.03	0.31 ± 0.02
	MAE	0.13 ± 0.03	0.15 ± 0.02	0.18 ± 0.08
Subject 5	R	0.94 ± 0.02	0.96 ± 0.01	0.92 ± 0.2
	RMSE	0.21 ± 0.03	0.16 ± 0.04	0.25 ± 0.02
	MAE	0.16 ± 0.01	0.11 ± 0.03	0.18 ± 0.08
Subject 6	R	0.93 ± 0.02	0.92 ± 0.01	0.92 ± 0.01
	RMSE	0.24 ± 0.04	0.30 ± 0.03	0.26 ± 0.08
	MAE	0.11 ± 0.02	0.17 ± 0.01	0.17 ± 0.07
Subject 7	R	0.91 ± 0.01	0.92 ± 0.01	0.88 ± 0.04
	RMSE	0.25 ± 0.04	0.027 ± 0.02	0.37 ± 0.03
	MAE	0.14 ± 0.01	0.14 ± 0.01	0.17 ± 0.07
Subject 8	R	0.93 ± 0.02	0.92 ± 0.03	0.93 ± 0.01
	RMSE	0.25 ± 0.01	0.22 ± 0.05	0.21 ± 0.07
	MAE	0.09 ± 0.02	0.13 ± 0.05	0.16 ± 0.07
Subject 9	R	0.87 ± 0.02	0.87 ± 0.01	0.84 ± 0.02
	RMSE	0.42 ± 0.05	0.42 ± 0.03	0.46 ± 0.02
	MAE	0.16 ± 0.02	0.23 ± 0.03	0.16 ± 0.065
Mean R		0.89 ± 0.09	0.91 ± 0.06	0.85 ± 0.12
Mean RMSE		0.28 ± 0.11	0.28 ± 0.09	0.35 ± 0.03
Mean MAE		0.14 ± 0.05	0.16 ± 0.05	0.15 ± 0.05

While analyzing the subject-wise results, we observed a good performance (*R* > 0.90) that is achieved almost throughout the 9 subjects. The two exceptions were the performance of the models regarding subjects 2 and 9. While subject 9 slightly underperformed with a correlation coefficient of *R* = 0.87, subject 2 failed to perform a similar result. The metrics for subject 2 resulted in a correlation coefficient *R* = 0.64, *RMSE* = 0.51, and *MAE* = 0.25 for the CNN on raw features when compared to other subjects, such as subject 3 with a result of *R* = 0.96, *RMSE* = 0.15, and *MAE* = 0.07.

In the overall results, after taking the average over the 9 subjects, we portrayed a similar scenario as observed in section Speed-Wise Evaluation. For example, we experienced the slightly inferior performance of the features in the frequency domain while the other two feature sets had a similar performance. The CNN on raw features [*R* = 0.89 ± 0.09, *RMSE* = 0.28 ± 0.11, *MAE* = 0.14 ± 0.05] and the LSTM on time-domain [*R* = 0.91 ± 0.06, *RMSE* = 0.28 ± 0.09, *MAE* = 0.16 ± 0.05] outperformed the CNN on frequency domain [*R* = 0.85 ± 0.12, *RMSE* = 0.35 ± 0.03, *MAE* = 0.15 ± 0.05] in all the metrics. It is important to mention that the relatively high SD resulted from the inferior performance regarding subject 2. To summarize, we observed that the CNN on frequency domain features performed poorly for almost every subject when compared to the CNN on raw features and the LSTM on time-domain features. The CNN on raw features and the LSTM on time-domain features were observed to show similar results.

## Discussion

This study was aimed to examine the effectiveness of ML models in estimating ankle joint power through FMG data and proposed a correct combination of features set and model for better estimation of ankle joint power. In this study, only intra-subject testing was evaluated, because FMG signal varies from subjects due to individual anatomy. Hence, inter-subject testing was expected to be ineffective. Therefore, the focus of this study was set on intra-subject testing. As expected and already shown in Barua et al. (2021), the DL models delivered a more effective performance in the estimation of ankle joint power. Especially the CNN model was able to deliver good performances using only 8 raw features. The strength of the CNN model is the high feature extraction by filters. Therefore, it hardly requires any additional feature extraction methods. However, to reach the level of similar performance as CNN did, more features had to be extracted with the other ML models.

**Figure 7:**
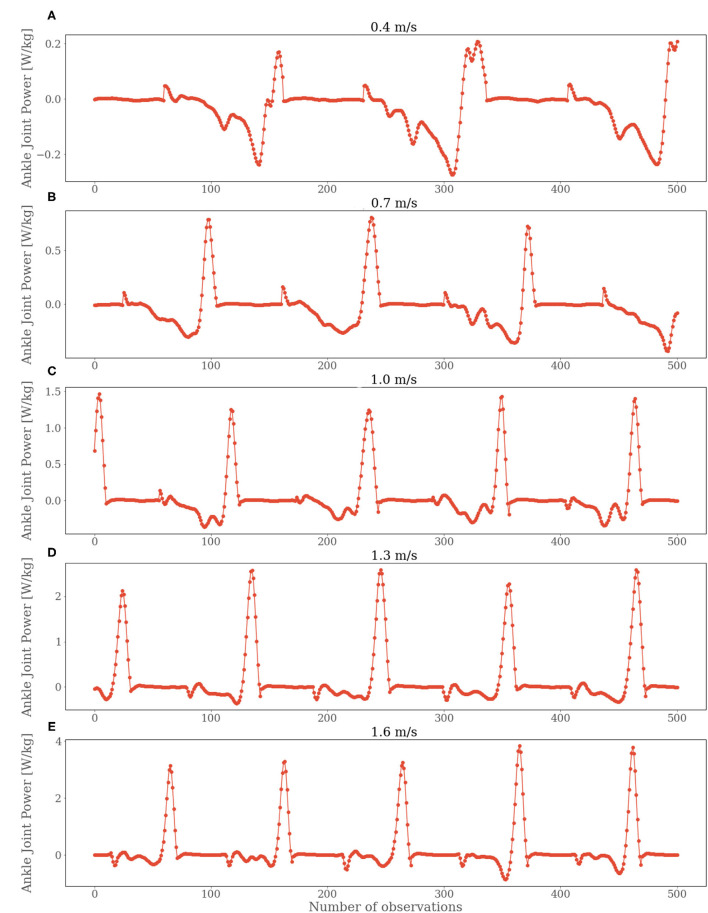
Ankle joint power values for subject 2 for all the velocities. Starting from 0.4 m/s in **(A)**, 0.7 m/s in **(B)**, 1.0 m/s in **(C)**, 1.3 m/s in **(D)** and 1.6 m/s in **(E)**.

The time-domain features improved the performances for both the CBR and LSTM models. However, time-domain features barely enhanced the predictive performance of CNN. On the other hand, the frequency-domain features could not improve the performance of any model we used. Although the models were able to deliver decent performance for high velocities with frequency-domain features, the outcome was comparatively very poor for low velocities. It signifies that the chosen frequency features by an FFT were not the correct choice for estimating ankle joint power using FMG signals. Nevertheless, it may still be possible that other frequency-domain features could improve the performance and can be explored in future works. From the results presented above, it can be perceived that the CNN slightly outperformed the other models for slower velocities. However, other challenge of this study was to find a suitable model that could deliver an acceptable performance throughout all the velocities. The inferior results for the slower velocities were expected since the same scenario was observed in a previous study (Jiang et al., 2019). However, they were able to shrink the performance gap in correlation coefficient for lower and higher velocities to 0.06. In our case, the difference was about 0.20 or higher. A possible explanation for this scenario is that the peak ankle joint power for slower speeds was lower than for higher speeds. Moreover, the ankle joint power for slower speeds has more oscillations, as can be seen in Figure 7. In brief, this study signifies that estimating ankle joint power using FMG signals is not as effective as using IMUs. Still, with a proper combination of ML models and feature sets, a considerable performance can be acquired using FMG signals.

**Figure 8:**
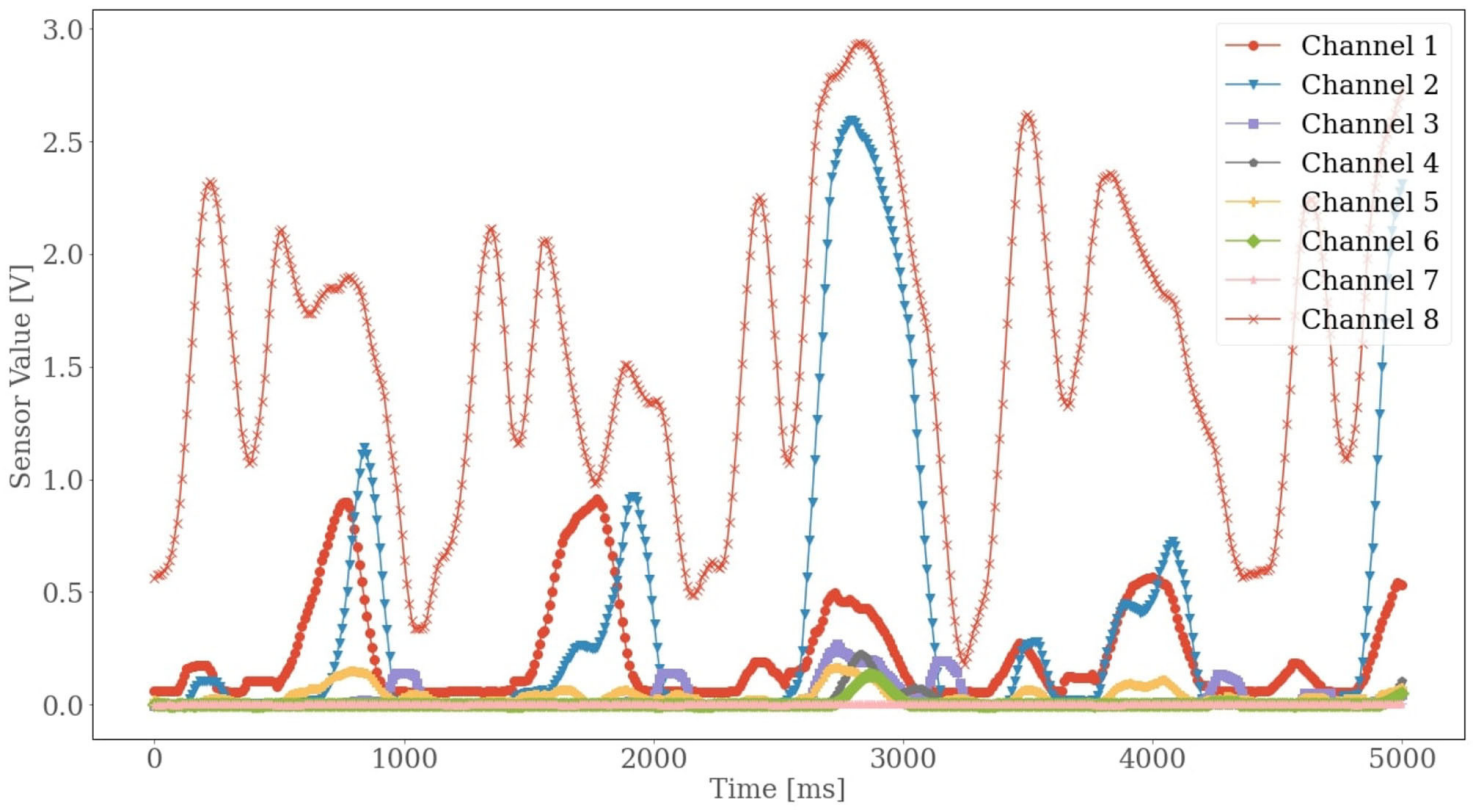
Smoothed sensor values for subject 2 for the velocity of 1.6 m/s.

In addition, we observed slight variations in performance for different subjects. Particularly for subject 2, the variation was noticeable. All the models performed poorly for subject 2. To investigate the reason behind such behavior, we did some statistical and graphical investigation. We found that the data accumulated for subject 2 contained many outliers in true ankle joint power. Moreover, the FMG signals for subject 2 seemed to have lower values than they had for other subjects (Figure 8). Comparing the results from our study with the results from the study (Jiang et al., 2019), we realize that the IMU is better suited for solving the problem of estimating the ankle joint power, especially because of the higher correlation coefficient (*R* = 0.98 ± 0.01) and lower RMSE (0.06 ± 0.01) using IMUs. The measuring unit in the IMUs (acceleration and angular velocity for the three-axis) seemed to work as better predictors for estimating ankle joint power. Besides, two IMUs were used on two distinct leg positions in the previous study (Jiang et al., 2019), while this study used only one FMG strap on one leg position to collect data. In the future, a similar study can be conducted to see with more FMG straps on more body positions to explore if it can help to improve the models' predictive performance as it did for other studies (Sakr and Menon, 2017). Another option for future work is to focus on the signal processing side. A more profound finetuning of DL models or combined feature sets may help to increase the predictive accuracy. Finally, a study can also be conducted to observe if our proposed approach is feasible for other datasets in future work.

## Conclusion

Since the computation of ankle joint power plays a significant role in various fields, such as health care or sports athletes' analyses, estimating ankle joint power with a simple model can make the process more accessible. We explored how effective FMG data from young, healthy subjects can be in predicting ankle joint power with proper ML models and feature sets.

The results we acquired from the study showed that we could generate a considerably effective LSTM model based on the time-domain features to achieve a correlation coefficient of over *R* = 0.91 ± 0.07 and CNN model using raw features to achieve a correlation coefficient of over *R* = 0.89 ± 0.13, which were acceptable when compared other similar studies (Jiang et al., 2019; Miyashita et al., 2019; Barua et al., 2021). However, the results were only valid for intra-subject testing. We assume that the result will not be similarly effective for inter-subject evaluation since the FMG sensor values seemed to change from subject to subject due to their limb size, skin thickness, and muscle density, even among healthy patients (Xiao and Menon, 2019).

Another focus of this study was whether or not the FMG data could estimate ankle joint power for slow walking speeds. It would primarily foster gait analyses for older people. Nonetheless, we could not overcome the performance gap between slow and fast walking speeds despite using various feature extraction methods and ML algorithms. Besides, FMG signals were not as effective as IMUs for such applications.

As mentioned earlier, in the future, studies may take place to explore the effectiveness of using more FMG straps on different body positions and the potency of other ML models and more robust feature sets to build a more functional system for more accurate estimation of ankle joint power.

## Data Availability Statement

The datasets presented in this article are not readily available because the Office of Research Ethics at Simon Fraser University did not provide approval for them to be shared publicly. Requests to access the datasets should be directed to CM, carlo.menon@hest.ethz.ch.

## Ethics Statement

The studies involving human participants were reviewed and approved by the Office of Research Ethics at Simon Fraser University. The patients/participants provided their written informed consent to participate in this study.

## Author Contributions

OH, AB, XJ, and CM: conceptualization and writing-review and editing. XJ and CM: data curation and supervision. OH and AB: formal analysis and methodology. XJ: funding acquisition. OH: investigation and writing an original draft. All authors have read and agreed to the published version of the manuscript.

## Funding

This research was supported by the Natural Sciences and Engineering Research Council of Canada (NSERC), the Canadian Institutes of Health Research (CIHR), and the Canada Research Chair (CRC) program. Open access funding was provided by ETH Zurich.

## Conflict of Interest

The authors declare that the research was conducted in the absence of any commercial or financial relationships that could be construed as a potential conflict of interest.

## Publisher's Note

All claims expressed in this article are solely those of the authors and do not necessarily represent those of their affiliated organizations, or those of the publisher, the editors and the reviewers. Any product that may be evaluated in this article, or claim that may be made by its manufacturer, is not guaranteed or endorsed by the publisher.
