# Erratum: Building effective machine learning models for ankle joint power estimation during walking using FMG sensors

**DOI:** 10.3389/fnbot.2023.1187128

**Published:** 2023-03-28

**Authors:** Frontiers Production Office

**Affiliations:** Frontiers Media SA, Lausanne, Switzerland

**Keywords:** gait analysis, FMG, ankle joint power, machine learning, LSTM, CNN

An omission to the funding section of the original article was made in error. The following sentence has been added: “Open access funding was provided by ETH Zurich”.

The original article has been updated.

